# Inaccurate Diagnosis of HIV-1 Group M and O Is a Key Challenge for Ongoing Universal Access to Antiretroviral Treatment and HIV Prevention in Cameroon

**DOI:** 10.1371/journal.pone.0007702

**Published:** 2009-11-06

**Authors:** Avelin F. Aghokeng, Eitel Mpoudi-Ngole, Henriette Dimodi, Arrah Atem-Tambe, Marcel Tongo, Christelle Butel, Eric Delaporte, Martine Peeters

**Affiliations:** 1 Virology Laboratory IMPM/IRD, Yaoundé, Cameroon; 2 UMR145, Institut de Recherche pour le Développement (IRD), University of Montpellier 1, Montpellier, France; McGill University Health Center, Montreal Chest Institute, Canada

## Abstract

**Background:**

Increased access to HIV testing is essential in working towards universal access to HIV prevention and treatment in resource-limited countries. We here evaluated currently used HIV diagnostic tests and algorithms in Cameroon for their ability to correctly identify HIV infections.

**Methods:**

We estimated sensitivity, specificity, and positive and negative predictive values of 5 rapid/simple tests, of which 3 were used by the national program, and 2 fourth generation ELISAs. The reference panel included 500 locally collected samples; 187 HIV -1 M, 10 HIV-1 O, 259 HIV negative and 44 HIV indeterminate plasmas.

**Results:**

None of the 5 rapid assays and only 1 ELISA reached the current WHO/UNAIDS recommendations on performance of HIV tests of at least 99% sensitivity and 98% specificity. Overall, sensitivities ranged between 94.1% and 100%, while specificities were 88.0% to 98.8%. The combination of all assays generated up to 9% of samples with indeterminate HIV status, because they reacted discordantly with at least one of the different tests. Including HIV indeterminate samples in test efficiency calculations significantly decreased specificities to a range from 77.9% to 98.0%. Finally, two rapid assays failed to detect all HIV-1 group O variants tested, with one rapid test detecting only 2 out of 10 group O specimens.

**Conclusion:**

In the era of ART scaling-up in Africa, significant proportions of false positive but also false negative results are still observed with HIV screening tests commonly used in Africa, resulting in inadequate treatment and prevention strategies. Depending on tests or algorithms used, up to 6% of HIV-1 M and 80% of HIV-1 O infected patients in Cameroon do not receive ART and adequate counseling to prevent further transmission due to low sensitivities. Also, the use of tests with low specificities could imply inclusion of up to 12% HIV negative people in ART programs and increase budgets in addition to inconveniences caused to patients.

## Introduction

Needs for HIV/AIDS care and treatment are increasing continuously in resource-poor settings, especially in Africa where the majority of people infected with HIV live. In addition to provide treatment to HIV infected individuals, the World Health Organization (WHO) and the Joint United Nations Programme on HIV/AIDS (UNAIDS) recommend also that prevention of new HIV transmissions should be considered as a key element of the global strategy of fight against HIV/AIDS [1]. A recent study even suggests that universal voluntary HIV testing and immediate antiretroviral treatment (ART), combined with present prevention approaches, could have a major effect on severe generalised HIV/AIDS epidemics [2]. However, efficient implementation of these prevention and care strategies require correct identification of non-infected and infected people.

Because they were historically developed based on HIV-1 subtype B prototype strains, HIV tests initially showed limitations to detect HIV-1 group O [3], but also some group M variants, especially during the serological window period [4]. Considerable efforts have been made to improve the performance of these assays. Inclusion of HIV-1 group O antigens or the use of broadly cross-reactive antigens reduced limitations related to the high genetic diversity of HIV [5] and the simultaneous detection of HIV antigens (p24) and anti-HIV antibodies by fourth generation assays reduced the window period [6], [7]. Despite these efforts, the performance of certain serological assays is still suboptimal as illustrated by some studies [8]–[10] and the high rate of HIV indeterminate results generated by HIV serological tests remains a major concern in African countries [11]–[13].

Current ART scale-up in developing countries is increasing the demand for HIV testing, especially through decentralized services in peripheral areas where lack of human resources and laboratory infrastructures are very common. For these reasons, there has been a proliferation of new rapid tests produced by several companies around the world, which must obtain the US Food and Drug Administration and the European Community label before introduction in US and Europe respectively, but are not subjected to any regulation prior to use in African countries, where there are generally selected and ordered by national programs based on cost and not on their performance. There is thus a need to monitor quality and reliability of these new tests. Cameroon is a country with an extreme high HIV genetic diversity, where approximately all known HIV-1 group M variants co-circulate, but also the more divergent HIV-1 group O and N viruses [14], [15]. Because of this high genetic diversity, HIV diagnosis in Cameroon is challenging and continues to require a specific attention.

In this report, we evaluated the performance of three HIV rapid assays recently introduced and recommended as first line tests in Cameroon by the government. They were selected because of their lower cost and without any prior evaluation on a local serum panel. We also reassessed the performance of two other rapid assays that we evaluated six years ago in Cameroon and which are still used in certain settings, and two fourth generation ELISAs, frequently used as reference assays in the region. Our study illustrates that despite the ongoing scale-up of ART in Africa, a significant proportion of false positive and also false negative results are observed with HIV screening tests locally used.

## Materials and Methods

### Reference Sample Panel

Between July and December 2007, we constituted a plasma panel by using discarded blood units from the blood bank of the Central Hospital in Yaoundé, the capital city of Cameroon. According to the local recommendations, blood and blood products intended for medical use, as transfusion, should be free of antibodies to HIV, hepatitis C virus (HCV), hepatitis B virus (HBV), and syphilis infections. Therefore, we collected from the blood bank all blood units labeled as “discarded” due to presence of at least one of these pathogens to constitute the reference sample panel. A total of 490 anonymous samples tested by the blood bank as HIV positive (n = 241) or HIV negative (n = 249) were collected. The initial HIV status of these samples was based on the results of HIV assays performed by the blood bank which were variable during this time period and included the Genscreen HIV-1/HIV-2 plus (Bio-Rad, Hercules, CA), HIV-1+2 Ag/Ab (Fortress Diagnostics Limited, Antrim, UK), HIV(1+2) Rapid Test Strip (KHB Shanghai Kehua Bioengineering Co. Ltd), and Determine HIV-1/2 (Inverness Medical Innovations Inc., Waltham, MA). Upon arrival at the Virology Laboratory IMPM/IRD, (Yaoundé, Cameroon), plasma was aliquoted for all samples and stored at −20°C and a buffy-coat layer, containing high concentrations of leukocytes, was also collected from each sample and stored at −20°.

We also constituted a specific HIV-1 group O panel comprising 10 left over specimens of previously identified group O infected patients confirmed by PCR and sequence analysis. All specimens of this HIV-1 group O panel were collected less than one year prior to the evaluation and only vials that were never thawed in the past were used for the present evaluation.

### Serological Testing and Evaluation of the Different HIV Serological Assays

All the plasma samples of the reference panel were tested in parallel with 5 HIV rapid tests and 2 fourth generation ELISAs, all commercially available in Cameroon. Table 1 summarizes the characteristics of all serological tests used. The rapid tests used included: Retrocheck HIV (Qualpro Diagnostics, Goa, India), SD Bioline HIV 1/2 3.0 (Standard Diagnostics Inc., Kyonggi-do, South Korea), HIV(1+2) Rapid Test Strip (KHB Shanghai Kehua Bioengineering Co. Ltd), Determine HIV-1/2 (Inverness Medical Innovations Inc., Waltham, MA), and ImmunoComb II HIV 1&2 BiSpot (Orgenics LTD., Yavne, Israel). The two fourth generation ELISAs, detecting simultaneously anti-HIV antibodies and HIV antigens, were Enzygnost HIV Integral II (DADE BEHRING, Marburg, Germany) and Murex HIV Ag/Ab-Combination (Murex Biotech Ltd, Kent, UK). Two rapid tests, Determine and ImmunoComb II, were undergoing a reevaluation six years after their first evaluation in Cameroon [11], while the three others were assessed for the first time in Cameroon. A line immunoassay, Inno-Lia HIV I/II Score (Innogenetics, Gent, Belgium), was used as a confirmatory assay. All assays were performed according to the manufacturers' instructions.

**Table 1:** Characteristics of the HIV diagnostic assays evaluated as described by manufacturers.

Test name	Manufacturer	Assay type	Antibody and antigen used	Sample type	Local price ($)
*Simple/rapid*					
Retrocheck HIV	Qualpro Diagnostics, Goa, India	Immunochromatographic assay	HIV1 (gp41, p24) and HIV2 (gp36) recombinant proteins	Serum/plasma/whole blood	0.8 $
SD Bioline HIV 1/2 3.0	Standard Diagnostics Inc., Kyonggi-do, South Korea	Immunochromatographic assay	HIV1 (gp41, p24) and HIV2 (gp36) recombinant proteins	Serum/plasma/whole blood	1.9 $
HIV(1+2) Rapid Test Strip	KHB Shanghai Kehua Bioengineering Co. Ltd	Immunochromatographic assay	HIV-1 and HIV-2	Serum/plasma/whole blood	0.7 $
Determine HIV-1/2	Inverness Medical Innovations Inc., Waltham, MA	Immunochromatographic assay	Recombinant and synthetic peptides	Serum/plasma/whole blood	1.1 $
ImmunoComb II HIV 1&2 BiSpot	Orgenics LTD., Yavne, Israel	Dot immunoassay	HIV-1and HIV-2 synthetic peptides	Serum/plasma	2.4 $
*ELISA*					
Enzygnost HIV Integral II	DADE BEHRING, Marburg, Germany	Sandwich ELISA	HIV-1 gp41, O gp41, and HIV-2 gp36 proteins and peptides	Serum/plasma	4.5–5 $
Murex HIV Ag/Ab-Combination	Murex Biotech Ltd, Kent, UK	Sandwich ELISA	HIV-1 Env and Pol, HIV-2 Env, HIV-1 O recombinant proteins	Serum/plasma	4.5–5 $
*LIA*					
l'Inno-Lia HIV I/II Score	Innogenetics, Gent, Belgium	Blot	Recombinant proteins and synthetic peptides	Serum/plasma	40–45 $

Samples that scored negative in all 7 screening assays (rapid tests and ELISAs) were considered as HIV negative. Samples positive to all screening assays were classified as HIV positive. Samples with discordant results among the different rapid tests and ELISAs were further tested with the confirmatory assay, Inno-Lia HIV, and classified based on the confirmation result. Specimens that reacted discordantly with screening assays and were neither confirmed as positive nor as negative with the confirmatory test were considered HIV indeterminate, and were further tested with a highly sensitive polymerase chain reaction (PCR) to detect present of HIV proviral DNA as mentioned below. All HIV positive samples from the reference panel were subjected to an *in-house* V3-loop peptide ELISA to discriminate between HIV-1 group M, O, and N, and HIV-2 as described previously [16], [17].

### Confirmatory PCR Analyses

PCR testing was done on all samples that were considered as HIV indeterminate because they reacted discordantly with the screening assays and were not successfully confirmed as HIV positive or negative using the Inno-lia confirmatory assay. Proviral DNA was extracted from uncultured peripheral blood mononuclear cells (PBMCs) contained into the collected buffy-coat layers using the QIAamp DNA Blood mini kit (Qiagen, Courtaboeuf, France) as manufacturer's instruction recommends. For PCR testing, several sets of previously described universal and highly sensitive primers known to amplify a large variety of HIV and SIV strains in the *pol* region were used [18]–[20].

### Statistical Analyses

The performance of the assays was expressed in terms of sensitivity, specificity, efficiency, and negative and positive predictive values. The 95% confidence intervals (CI) of the estimated sensitivities, specificities and efficiency were also calculated. For performance determination and CI calculation, previously described formulas were used [11]. Negative and positive predictive values (NPV and PPV) were determined based on 5.5% HIV prevalences reported in 2004 from the last population based survey in Cameroon [21]. The default scenario when determining the performance of the different assays excluded samples with indeterminate results as recommended by WHO [22], but in addition we also estimated the performance with these samples included, and in this case, we assumed that they represent negative samples because of the absence of HIV proviral DNA detection by PCR, and therefore most likely represent false positive reactivities. Finally, we evaluated the performance of the national HIV testing algorithm in use in Cameroon at the time of the assays evaluation.

### Ethical Considerations

The study was considered as a routine program evaluation and quality monitoring of HIV diagnosis and the Cameroonian Ministry of Health, which co-funded the study, do not recommend any ethics approval in such conditions, especially when no human subject is involved. All samples were anonymously obtained from uncorrelated-discarded blood units to develop the reference sample panel and no human experimentation was conducted.

## Results

### Characteristics of the Reference Sample Panel

A total of 490 plasma samples, collected at the blood bank, were tested in parallel using 5 simple rapid assays and 2 fourth generation ELISAs. 259 samples were confirmed as HIV negative because they scored negative with all the 7 screening assays (n = 204) or reacted discordantly with at least one assay but were further confirmed negative with the Inno-Lia HIV confirmatory test (n = 55). Similarly, 187 samples were confirmed HIV positive because they scored positive to all the 7 screening assays (n = 177) or displayed discordant results with the screening assays and were further confirmed positive with the Inno-Lia HIV confirmatory assay (n = 10). A total of 44 samples representing 9% of our sample panel, were considered as HIV indeterminate, because of discordant results among the 7 screening assays and lack of criteria to identify HIV positivity or negativity in the confirmatory Inno-Lia test. Interestingly, all attempts to identify proviral HIV DNA in these 44 indeterminate samples using universal and broadly sensitive PCR primers were unsuccessful. Analyses of positive samples (n = 187) with the discriminatory V3-loop peptide ELISA identified all as HIV-1 group M variants.

### Performance of Evaluated HIV Screening Assays to Detect HIV-1 Group M Variants

Two distinct scenarios were used to determine the performance of the evaluated assays. We first excluded samples with an indeterminate HIV serology to comply with WHO guidelines when evaluating HIV screening assays, and considered only samples for which we obtained a definite HIV negative or positive status. A total of 446 samples, including 187 HIV positive and 259 HIV negative where considered in this first scenario to calculate sensitivity, specificity, efficiency, and PPV and NPV. However, based on our previous experience and reports on the relative high proportions of indeterminate results of HIV serological screening assays in Africa, we also evaluated the performance of the assays including indeterminate results. When included, we considered these indeterminates (n = 44) as negative since all attempts to confirm HIV infection with molecular assays were unsuccessful. The total sample size for the second scenario was 490, with 187 HIV positive and 303 HIV negative.

Among the 5 rapid tests, only 2, Determine and ImmunoComb II displayed 100% (187/187) sensitivity. Retrocheck showed the lowest sensitivity, 94.1% (176/187) only, since 11 HIV-1 group M positive samples out of 187 were not detected. SD-Bioline and HIV(1+2) Strip each missed 5 positive HIV-1 group M specimens and showed a final sensitivity of 97.3% (182/187) (Table 2). None of the 2 ELISAs had 100% sensitivity; Enzygnost missed 1 positive specimen and consequently showed a sensitivity of 99.5% (186/187) while Murex showed a sensitivity of 98.9% (185/187) because it failed to detect 2 positive HIV-1 group M specimens (Table 2). Assays sensitivities were not affected by addition of indeterminate samples since we included these samples as HIV negatives (Table 3).

**Table 2:** Performance of HIV assays evaluated, indeterminate result samples excluded.

HIV assay	Totalα	HIV positive	HIV negative	True positive	False negative	True negative	False positive	Sensitivity % (95% CI)β	Specificity % (95% CI)	Efficiency % (95% CI)	PPV % (5.5%)δ	NPV % (5.5%)
*Simple/rapid*												
Retrocheck HIV	446	187	259	176	11	255	4	94.1 (89.8–96.7)	98.5 (96.1–99.4)	96.6 (94.5–98.0)	78.1	99.7
SD Bioline HIV 1/2 3.0	446	187	259	182	5	240	19	97.3 (93.9–98.9)	92.7 (88.8–95.3)	94.6 (92.1–96.4)	43.6	99.8
HIV(1+2) Rapid Test Strip	446	187	259	182	5	256	3	97.3 (93.9–98.9)	98.8 (96.6–99.6)	98.2 (96.5–99.1)	83.0	99.8
Determine HIV-1/2	446	187	259	187	0	228	31	100.0 (98.0–100.0)	88.0 (83.5–91.4)	93.0 (90.3–95.1)	32.7	100.0
ImmunoComb II HIV 1&2	446	187	259	187	0	232	27	100.0 (98.0–100.0)	89.6 (85.3–92.7)	93.9 (91.3–95.8)	35.8	100.0
*ELISAs*												
Enzygnost HIV Integral II	446	187	259	186	1	255	4	99.5 (97.0–99.9)	98.5 (96.1–99.4)	98.9 (97.4–99.5)	79.9	99.9
Murex HIV Ag/Ab-Combo	446	187	259	185	2	249	10	98.9 (96.8–99.7)	96.1 (93.0–97.9)	97.3 (95.4–98.5)	59.9	99.9

α Total number of samples included in the calculations.

β 95% confidence intervals.

δ General population HIV prevalence reported in Cameroon in 2004.

**Table 3:** Performance of HIV assays evaluated, indeterminate result samples included.

HIV assay	Totalα	HIV positive	HIV negative	True positive	False negative	True negative	False positive	Sensitivity % (95% CI) β	Specificity % (95% CI)	Efficiency % (95% CI)	PPV % (5.5%)δ	NPV % (5.5%)
*Simple/rapid*												
Retrocheck HIV	490	187	303	176	11	297	6	94.1 (89.8–96.7)	98.0 (95.7–99.1)	96.5 (94.5–98.0)	73.5	99.7
SD Bioline HIV 1/2 3.0	490	187	303	182	5	275	28	97.3 (93.9–98.9)	90.8 (87.0–93.5)	93.3 (90.7–95.2)	38.0	99.8
HIV(1+2) Rapid Test Strip	490	187	303	182	5	298	5	97.3 (93.9–98.9)	98.4 (96.2–99.3)	98.0 (96.3–98.9)	77.4	99.8
Determine HIV-1/2	490	187	303	187	0	236	67	100.0 (98.0–100.0)	77.9 (72.9–82.2)	86.3 (83.0–89.1)	20.8	100.0
ImmunoComb II HIV 1&2	490	187	303	187	0	239	64	100.0 (98.0–100.0)	78.9 (73.9–83.1)	86.9 (83.7–89.6)	21.6	100.0
*ELISAs*												
Enzygnost HIV Integral II	490	187	303	186	1	273	30	99.5 (97.0–99.9)	90.1 (86.2–93.0)	93.7 (91.2–95.5)	36.9	99.9
Murex HIV Ag/Ab-Combo	490	187	303	185	2	284	19	98.9 (96.8–99.7)	93.7 (90.4–96.0)	95.7 (93.5–97.2)	47.9	99.9

α Total number of samples included in the calculations.

β 95% confidence intervals.

δ General population HIV prevalence reported in Cameroon in 2004.

Overall, specificities of the tests ranged from 88.0% (228/259) for Determine to 98.8% (256/259) for HIV(1+2) Strip for simple rapid tests. The fourth generation ELISAs displayed specificities of 96.1% (249/259) for Murex and 98.5% (255/259) for Enzygnost (Table 2). Inclusion of samples with indeterminate results dramatically affected these specificities for all tests. Indeed, since we included these samples as negatives, assays with lowest specificity were significantly affected because they tend to generate high levels of false positive results. The most affected rapid tests were Determine, which showed a specificity of only 77.9% (236/303) in this scenario, ImmunoComb with 78.9% (239/303), and SD Bioline with 90.8% (275/303). The two ELISAs were also strongly affected with inclusion of indeterminate results and showed specificities of 90.1% (273/303) and 93.7% (284/303) respectively (Table 3).

As a consequence of the low specificities, the overall assay efficiencies were also low, ranging between 93.9% (415/446) for Determine and 98.2% (438/446) for HIV(1+2) Strip for rapid tests. The two ELISAs also showed relatively low efficiency, 98.9% (441/446) for Enzygnost and 97.3% (434/446) for Murex (Table 2). As expected, inclusion of indeterminate results negatively affected the efficiency of all assays evaluated because of the decrease in specificities (Table 3). Although we observed good NPV for all the assays, between 99% and 100%, obtained PPV were very low in both scenarios, with and without indeterminate results (Table 2 & 3).

### Detection of HIV-1 Group O Samples

We assessed the ability of the 7 screening assays to detect HIV-1 group O infection which represents about 1% of HIV-1 infections in Cameroon [14], [17]. Although we used only 10 HIV-1 O positive samples, results obtained helped to identify assays capable to detect this HIV-1 variant. The two ELISAs and three rapid tests, Determine, ImmunoComb II, and SD Bioline correctly identified all group O specimens. However, HIV(1+2) Strip missed 2 positive samples while Retrocheck missed 8 samples out of 10 tested.

### Performance of the Two Most Commonly Used National HIV Testing Algorithms in Cameroon

At the time of this evaluation, the national strategy in use in Cameroon for routine HIV diagnosis included two consecutive rapid tests; Retrocheck being used as the first screening test and SD Bioline to confirm reactive specimens with the first test. This algorithm was applied on the reference panel of HIV-1 group M samples to determine its performance in terms of sensitivity and specificity. As expected, the algorithm sensitivity was quite close to the sensitivity of the first screening assay, Retrocheck, with a value of only 94.7%. The specificity of the algorithm was also not satisfactory, since we obtained a value of 98.8%. In other words, results of the algorithm performance show that when people were tested in Cameroon using this strategy, 6 out of 100 persons were falsely declared HIV negative, and 2 out of 100 persons were falsely declared HIV positive. Just for illustration, 2% false HIV positive individuals in a high burden country as South Africa with more than 5 million HIV infections will correspond to about 100000 people falsely declare HIV positive. Moreover, it should be noted that this algorithm can only detect 2 HIV-1 group O variants out of 10, since the first assay used is Retrocheck. At the level of a population, these results can have a strong negative impact on the outcome of currently implemented HIV prevention, care, and treatment programs.

In addition, the other algorithm commonly used in Cameroon, which included Determine and ImmunoComb II in a serial approach, Determine being the first or screening assay, while ImmunoComb II was used to confirm positive and indeterminate results also showed poor results. In fact, this strategy correctly detected all HIV positive samples and showed sensitivity of 100%, but because of the reduced specificity of both rapid assays, the algorithm that combined the two rapid tests generated many false positive results with and overall specificity of 91.5%. Therefore, using this algorithm as a national testing strategy will reduce the chance of having false negative results, but will dramatically increase the number of people falsely tested positive, and perhaps referred for a treatment initiation although they are HIV negative. This latter could increase budget for ART treatment at government levels in addition to all other indirect costs related to antiretroviral treatment and disagreements caused to the patients.

## Discussion

Reliable HIV testing is a critical entry point to life-sustaining healthcare services for people living with HIV and AIDS including ART services and implementation of prevention strategies. Based on current WHO/UNAIDS estimates, more than 80% of people living with HIV in low and middle-income countries do not know that they are infected and current guidelines recommend new options to increase the provision of HIV testing as the so called “opt-out” approach to provider-initiated HIV testing and counseling [1]. However, quality monitoring and assessment of HIV testing in these settings is lacking, and very few is known about the proportion of people falsely declared positive or negative because of reduced performance of assays or inappropriate HIV testing and its impact on current prevention and treatment strategies in resources-constrained settings.

In resource limited countries, WHO recommends a testing algorithm without the expensive confirmatory tests like western blot or Inno-Lia. Ideally, the first assay should thus have a 100% sensitivity combined to a good specificity and the second test used to confirm the initial positive results should have a 100% specificity coupled to a good sensitivity. None of the ELISAs and only two out of five rapid assays (Determine and ImmunoComb II) here evaluated showed a 100% sensitivity, and none of the 7 assays has a 100% specificity. Even the WHO and UNAIDS recommendations which propose that HIV tests should have a sensitivity of at least 99% and a specificity of 98% are not reached by the assays from our study [1]. In addition, we here showed that certain tests, not only fail to identify HIV infection with divergent HIV-1 group O but also with common HIV-1 group M variants circulating in Africa. This is in particular the case for recently developed assays which are available at low costs in resource-limited countries like Retrocheck, HIV(1+2) Rapid Test Strip, etc… Sensitivity of HIV diagnostic tools has been considerably increased to reduce the window period from weeks to days with the fourth generation ELISAs, but our study shows that these tests also fail to detect some HIV-1 group M infections. On the other hand, we showed also problems with the specificity of assays intended for confirmation in resource limited settings where implementation of confirmatory assays like Western Blot or Innolia was very early proven to be challenging because of the required logistics and the high cost [22]. Evaluation of alternative HIV testing strategies that do not include western blot has clearly shown that adequate combination of rapid and/or ELISA tests can have similar efficacy and even better than the reference strategies using the western blot [23]. However, as the results of our study here show, highly specific rapid assays and ELISAs are becoming increasingly rare and developing an HIV testing algorithm that combines good sensitivity and specificity in resource-limited countries can be challenging. This was well illustrated by the performance of the algorithm including Determine and ImmunoComb II that we tested, which showed very good sensitivity, but a low specificity with an overall false-positive rate close to 10%. Similar findings were recently reported from the Democratic Republic of Congo where a Médecins Sans Frontières team found that the local HIV testing strategy using two rapid assays, Determine and UniGold HIV (Trinity Biotech, Wicklow, Ireland) generated up to 10.5% false positive results [24]. Although low HIV prevalence can alter the characteristics of diagnostic assays by increasing the proportion of false positives, these findings suggest that current rapid testing strategies in resource limited countries which frequently involves the use of two sequential rapid tests should be correctly evaluated before implementation by programs at the national level, not only to limit the chance of having false negative HIV results, but also to make sure that people declared as HIV positive, who are often directly referred to treatment initiation, are really HIV infected. Moreover, we showed that certain tests, like ImmunoComb II, previously found as having a good specificity had a significant decreased specificity over time, stressing the need for regular evaluations also for known tests. This correct evaluation of rapid-testing algorithms prior to use is essential in countries as Cameroon where, because of cost limitations, HIV confirmatory tests or additional testing by cheaper ELISAs are not routinely recommended before treatment initiation.

The second problem we here highlighted is the difficulty for decision/policy makers of AIDS programs to implement appropriate HIV testing policies in resource-poor countries. Good practices recommend that HIV assays intended for use in a country should be first evaluated on a serum panel from patients infected with local and contemporary HIV strains to measure their performance, but also for their operational characteristics as storage conditions, equipment required, ease of use, etc. [1]. Unfortunately, this scenario is rarely applied due to practical constraints, limited resources, inappropriate policies and absence of laboratory experts in policymaking and program planning. In practice, test kits are selected and ordered by government officials with no or low experience in HIV diagnosis and selection is often only based on the lower price and not also on tests efficacy. Lack of national quality control policies including regular reassessment of tests used in the country, is also a major concern [25]. Also, because of ineffective supply chain management, stock-outs are also frequent and as a consequence, when existing, HIV testing algorithms are not applied and testing strategies are based on assays available as illustrated by the blood bank in Cameroon where we collected the samples for our serum panel. Inaccuracy of HIV diagnosis in the field is also often related to the fact that the staff in the health care facility is not adequately trained, a high turn over of personnel, the use of tests after expiration dates, or shipment of tests in sub-optimal conditions. The responsibility of funding agencies is a concern too, because they require numerous financial and administrative conditions for money use, but they rarely require quality monitoring and assessment of implemented programs as key indicators to evaluate the appropriate use of fundings.

In conclusion, our results showed that HIV diagnosis is still a major challenge in Cameroon, a country with a general population HIV prevalence of 5.5%, ranging from 2% in the Nord Region to 8.6% and 8.7% in the East and Nord-West Regions respectively [21]. There is a need to implement guidelines and provide resources for the validation of national testing strategies for HIV diagnosis on a regularly basis over time. Our observations in field conditions in Cameroon and other African countries stress also the urgent need for continuous training of laboratory personnel or health care workers performing HIV testing as well as for implementation of quality control programs to improve the quality of results obtained with these basic HIV tests in health care centers. The presence of laboratory experts in program planning and policymaking could significantly improve quality of HIV diagnosis.
